# Emergency intensive care unit pharmacist’s intervention may reduce time to four-factor prothrombin complex concentrate administration: a retrospective study

**DOI:** 10.1186/s40780-020-00161-z

**Published:** 2020-04-03

**Authors:** Shoichi Imanaka, Tatsuro Kuwabara, Hiroto Ikeda, Yasufumi Miyake, Hiromi Enomoto, Tetsuya Sakamoto, Nobuhiro Yasuno

**Affiliations:** 1 Pharmaceutical Department, Teikyo University School of Medicine Hospital, 2-11-1 Kaga, Itabashi-Ku, Tokyo, 173-8605 Japan; 2 Department of Emergency Medicine, Teikyo University School of Medicine, 2-11-1 Kaga, Itabashi-Ku, Tokyo, 173-8605 Japan; 3 Laboratory of Hospital Pharmacy Sciences, Faculty of Pharma Science, Teikyo University, 2-11-1 Kaga, Itabashi-Ku, Tokyo, 173-8605 Japan

**Keywords:** Prothrombin complex concentrate, Pharmacist, Time reduction, Warfarin, Anticoagulation reversal

## Abstract

**Background:**

Four-factor prothrombin complex concentrate (4F-PCC) must be administered as soon as possible, and in our emergency intensive care unit (EICU), pharmacists are available on weekdays for consultation on expediting 4F-PCC administration. Although recent reports have described a reduction in time to 4F-PCC administration, few studies have addressed if this is because of EICU pharmacist’s intervention, and there are no such studies in Japan. Therefore, we aimed to examine whether EICU pharmacist’s intervention reduced time to 4F-PCC administration.

**Methods:**

This single-center retrospective cohort study was conducted from December 2017 to May 2019. We enrolled patients who received 4F-PCC due to major bleeding or requirement of urgent surgical/invasive procedures (*n* = 10). Patients were divided into two groups, namely, the intervention group (*n* = 5), in which EICU pharmacists consulted on weekdays, and the nonintervention group (*n* = 5), in which an intervention was not possible because of the absence of the EICU pharmacist.

**Results:**

The median time from patient presentation to the EICU to 4F-PCC administration (103 min vs. 111 min, *p* = 0.4) was similar between the two groups; however, the median time from 4F-PCC prescription ordering to administration was significantly shorter in the intervention group than in the nonintervention group (21 min vs. 60 min, *p* = 0.02).

**Conclusions:**

EICU pharmacist’s intervention improves the process from 4F-PCC prescription to administration and can reduce time to 4F-PCC administration.

## Background

In Japan, the four-factor prothrombin complex concentrate (4F-PCC; Kcentra®) became available in September 2017 to patients receiving warfarin therapy owing to major bleeding or requirement of urgent surgical/invasive procedures [1]. 4F-PCC must be administered as soon as possible because an early reduction in the prothrombin time-international normalized ratio (PT-INR) has been reported to reduce mortality due to major bleeding [2]. In recent years, there have been reports on the reduction of time to 4F-PCC administration [3–7]. However, in the emergency intensive care unit (EICU), during the time period between patient presentation to 4F-PCC administration, it is necessary to confirm medication histories, design the complicated prescription, and combine intravenous vitamin K but the optimal procedure for this remains unknown.

In the United States, there are position paper and American Society of Health-System Pharmacists (ASHP) guidelines, which standardize the role of emergency department (ED) pharmacists [8, 9]. Although Japan has a Certified Pharmacist for Emergency Medicine system, the roles of ED pharmacists have not been standardized compared to those overseas. Furthermore, in the emergency system in Japan, hospitals are classified into primary, secondary and tertiary, and patients are categorized into levels according to their severity acuity, then transferred to the hospital. Therefore, ED pharmacists work according to the roles of each hospital. Because Teikyo University School of Medicine Hospital is a tertiary emergency hospital, the likelihood of patients with severe conditions being brought in is extremely high. We therefore think it is one of the roles of EICU pharmacists to administer emergency medications more appropriately and quickly. Few studies determined the effects of ED pharmacist’s intervention, and there are no such studies in Japan.

At our EICU, pharmacists are available on weekdays and offer strategies to expedite 4F-PCC administration. Therefore, we evaluated whether EICU pharmacist’s intervention reduced time to 4F-PCC administration.

## Methods

### Methods

This single-center retrospective cohort study was conducted from December 2017 to May 2019 because our hospital stored 10 vials of 500 units in the pharmacy department and initiated its use in December 2017. Patients who received 4F-PCC owing to major bleeding or requirement of urgent surgical/invasive procedures were enrolled. Further, patient information was extracted using electronic medical records. Exclusion criteria were 4F-PCC administration outside EICU (e.g., neurosurgical ward), administration of oral anticoagulation therapy other than warfarin, and patients not administered 4F-PCC at the time of presentation to the EICU. Patients were divided into the intervention group, in which EICU pharmacists consulted during the weekday, and the nonintervention group, in which intervention was not possible because of the absence of the EICU pharmacist.

### EICU pharmacist’s intervention

**Fig. 1:**
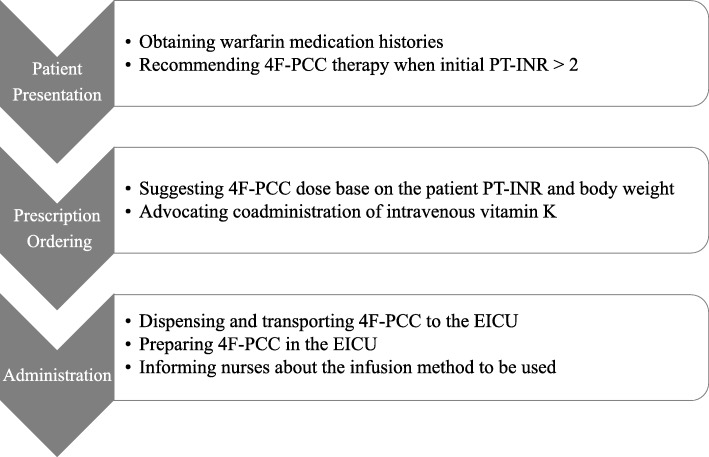
EICU pharmacist’s intervention. 4F-PCC, four factor prothrombin complex concentrate; EICU, emergency intensive care unit; PT-INR, prothrombin time-international normalized ratio

Pharmacists were available for consultation on weekdays at the EICU from Monday through Friday, excluding national holidays, from 8:00 am to 5:30 pm. The details of the EICU pharmacist’s intervention are as follows (Fig. 1): (1) obtaining warfarin medication history from the patient and the medication record book at the time of patient presentation to the EICU, (2) recommending 4F-PCC therapy to the emergency physician when initial PT-INR > 2, (3) suggesting 4F-PCC dose based on the patient PT-INR and body weight, (4) advocating coadministration of intravenous vitamin K, (5) dispensing and transporting 4F-PCC from the pharmacy department to the EICU, (6) preparing 4F-PCC in the EICU, and (7) informing nurses about the infusion method to be used.

### Data collection

We obtained data on patient background (age, gender, and body weight), type of injury, reason for administration, time from patient presentation to the EICU to 4F-PCC consent form acquisition, 4F-PCC dose, 4F-PCC infusion time, coadministration of intravenous vitamin K, diagnosis of disseminated intravascular coagulation (DIC), initial PT-INR, post-4F-PCC PT-INR, and time taken for 4F-PCC administration.

### Statistical analyses

The following parameters were compared between the intervention and nonintervention groups: (1) time from 4F-PCC prescription ordering to administration and (2) time from patient presentation to the EICU to 4F-PCC administration. All analyses were performed using JMP® pro 13 (SAS Institute Inc., Cary, NC, USA). Mann–Whitney U test was used to analyze continuous data, and Fisher’s exact test was used to analyze categorical data. A *p* value of < 0.05 was considered statistically significant.

## Results

Thirteen patients received 4F-PCC during the study period. Among them, three patients were excluded because 4F-PCC had been administered in the neurosurgical ward (*n* = 1), the patient was on oral anticoagulation other than warfarin (*n* = 1) and no 4F-PCC administration at the time of EICU presentation (*n* = 1). Therefore, we analyzed data from 10 patients, and Table 1 lists the survey contents. In nine of the 10 patients, the 4F-PCC dose was within ±10% of the manufacturer’s prescribed individualized dosing regimen, and one patient was underdosed (A). Intravenous vitamin K was coadministered in nine of the 10 patients, except in one patient (J). Although case G did not show an accurate value exceeding 3.84 in the clinical laboratory, all patients had PT-INR > 2 before 4F-PCC administration, and PT-INR decreased after 4F-PCC administration. Although DIC is contraindicated when 4F-PCC is administered, DIC was not observed at the time of presentation to the EICU in all patients. There were no differences in baseline characteristics between the two groups.

**Table 1:** 4F-PCC Administration Characteristics

No.	Age (Sex)	Median (IQR)	Body Weight (kg)	(kg, IQR)	4F-PCC dose (IU/kg)	Infusion time (min)	IV Vitamin K	initial PT-INR	post-4F-PCC PT-INR	Consent form acquistion time (min)	(min, IQR)	Time from patient presentation to prescription ordering (min)	Time from prescription ordering to administration (min)	Type of injury	Reason for administration
A	77(F)	74 (70–79)	55.5	55.5 (42.1–66.4)	18.0	8	+	2.20	1.32	105	103 (67–121)	105	5	ASDH	Bleeding
B	82(F)		38.0		26.0	8	+	3.69	1.39	103		87	16	Pelvic fracture	Bleeding
C	74(M)		46.2		54.1	12	+	11.98	1.42	137		105	39	Cholecystitis	Urgent procedures
D	68(F)		73.8		47.4	17	+	7.94	1.44	93		72	21	Sepsis	Urgent procedures
E	72(M)		59.0		25.4	24	+	3.94	1.37	41		17	24	Liver damage	Bleeding
F	89(F)	89 (67–92)	43.0	48.0 (42.5–63.0)	23.3	IV	+	2.71	1.26	177	45 (22–128)	46	157	ASDH	Bleeding
G	90(M)		73.0		24.0	17	+	3.84<	1.40	79		95	74	ICH	Bleeding
H	94(F)		48.0		25.0	19	+	2.95	1.15	45		60	51	Traumatic pneumothorax	Bleeding
I	71(F)		42.0		23.8	16	+	2.07	1.30	10		37	33	Pelvic fracture	Bleeding
J	63(M)		53.0		37.7	32	–	5.02	1.45	34		34	60	Pelvic fracture	Bleeding

*4F-PCC* four factor prothrombin complex concentrate; A-E: Intervention group F-J: Nonintervention group

*PT-INR* prothrombin time-international normalized ratio; *IQR* interquartile range;

*ASDH* acute subdual hemorrhage; *ICH* intracranial hemorrhage; *IV* intravenous

**Fig. 2:**
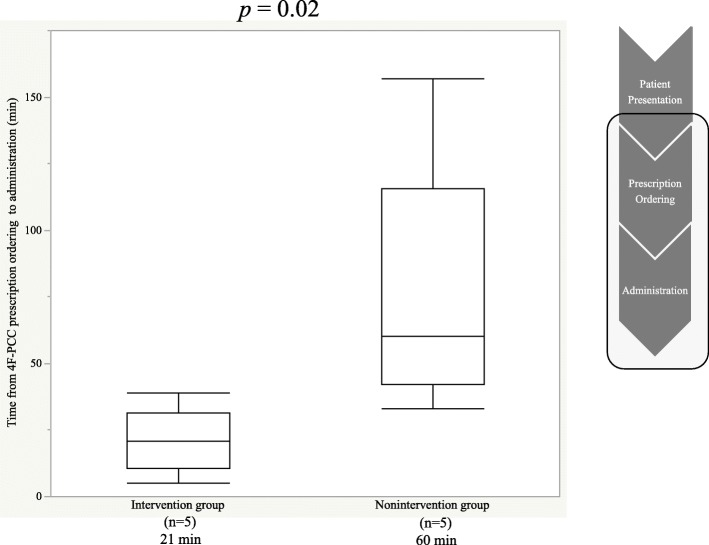
Median time from 4F-PCC prescription ordering to administration among study groups. 4F-PCC, four factor prothrombin complex concentrate

**Fig. 3:**
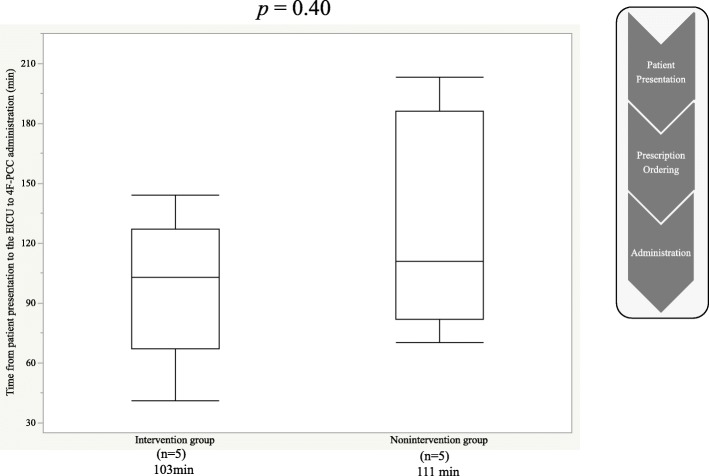
Median time from patient presentation to the EICU to 4F-PCC administration among study groups. EICU, emergency intensive care unit; 4F-PCC, four factor prothrombin complex concentrate

The median time from prescription ordering to administration was significantly reduced in the intervention group (21 min vs. 60 min, *p* = 0.02) compared with the nonintervention group (Fig. 2); however, median time from patient presentation to the EICU to 4F-PCC administration was similar between the two groups (103 min vs. 111 min, *p* = 0.4) (Fig. 3).

## Discussion

The effectiveness of 4F-PCC has been reported by studies in Japan and overseas [10–12], and reportedly, 4F-PCC administration shortens the time to PT-INR correction, especially when compared with conventional fresh frozen plasma [13]. Therefore, in our facility, we believe that it is important to administer 4F-PCC as soon as possible. Furthermore, we determined whether EICU pharmacist’s intervention could contribute to achieving both early and proper 4F-PCC administration by reducing the time to 4F-PCC administration based on ASHP guidelines [9].

All patients who were administered 4F-PCC had an initial PT-INR > 2, and the dosage provided was within ±10% of the recommended dosage in both groups, except in one patient. Intravenous vitamin K was administered to all patients in the intervention group but not to one of 5 patients in the nonintervention group. The infusion rate was 3 IU/kg/min or less according to the package insert. In addition, the infusion rate was maintained in all patients in the intervention group but not in one patient in the nonintervention group who received rapid intravenous infusion. Because 4F-PCC is very expensive, its proper use by pharmacists is necessary [14]. EICU pharmacists are involved in patient presentation to the EICU to 4F-PCC administration. Therefore, the following measures are being taken: (1) obtaining warfarin medication history and recommending 4F-PCC therapy to the emergency physician and (2) suggesting 4F-PCC dose and infusion rate and advocating coadministration of intravenous vitamin K. Naturally, the pharmacist in the pharmacy department can confirm the dosage in the electronic medical records. However, the time required for emergency physician to write information on the electronic records or time required for introducing a question to the prescribing emergency physician by telephone when there is a deficiency in prescription by the emergency physician becomes more necessary. Particularly, EICU pharmacists familiar with the 4F-PCC use can more reliably advocate the coadministration of intravenous vitamin K. Our results suggest that the EICU pharmacist’s intervention can contribute to the proper administration of 4F-PCC.

Regarding the time to 4F-PCC administration, the time from prescription ordering to administration was shorter by 39 min in the intervention group than in the nonintervention group. This reduction in time in the intervention group was probably because of the following reasons: (1) in the intervention group, 4F-PCC was stored in the pharmacy department rather than in the EICU, and cooperation between pharmacists in the EICU and the pharmacy department facilitated quick dispensation and transportation of 4F-PCC from the pharmacy to the EICU and (2) the EICU pharmacist confirmed the dose and promptly prepared 4F-PCC. It was suggested that the active involvement of the EICU pharmacist from the patient’s condition confirmation to the 4F-PCC administration in the immediate vicinity of the emergency physician in real-time greatly contributed. Similar to our study, studies in other countries have reported a decrease in time from prescription ordering to 4F-PCC administration owing to better pharmacy management and ED pharmacist’s intervention [4, 5].

In contrast, there was no difference between the two groups in the time taken from patient presentation to the EICU to 4F-PCC administration, possibly because of the following factors: (1) consent form acquisition time (103 min vs. 45 min, *p* = 0.29) was not different between the groups, although it took longer in the intervention group than in the nonintervention group and (2) because all patients received 4F-PCC after warfarin administration, it took a long time to determine PT-INR and body weight required for designing the prescription. However, Dalila et al. reported that a clinical pharmacist’s intervention decreased the time taken from patient presentation to the EICU to 4F-PCC administration by 140 min and shorter ICU length of stay [6]. Further, Jessica et al. determined that PT-INR ≥ 2 and intracranial hemorrhage was confirmed on imaging but not on patient presentation [7]. These studies suggest that various factors, such as time required for prescription ordering (especially in terms of determining PT-INR and body weight) and a patient’s family relationship, must be considered.

As a limitation of this study, the number of medical professionals is often lower at night and on holidays than on weekdays. We considered the same in our hospital, especially at night. However, because EICU has a higher number of medical staff members than other wards according to facility standards, the impact is considered small. Thus, it is necessary to increase the deployment time of EICU pharmacists in our hospital because pharmacists are permanently stationed in EICUs for 24 h in some institutions in Japan [15]. Many hospitals overseas have protocols for emergency warfarin reversal [16], and similarly, some hospitals in Japan have in-house guidelines for proper use [17]. Therefore, we need to develop a protocol for appropriate 4F-PCC administration, conduct workshops, and reduce time to 4F-PCC administration in the absence of EICU pharmacists.

We could not find any clinical outcome in this study. Because the time from prescription ordering to 4F-PCC administration was reduced by the intervention of the EICU pharmacist, further investigation of the factors contributing to the shortening of the time from patient presentation to the EICU to 4F-PCC administration may lead to shortening the ICU length of stay in Japan. Therefore, we hope to achieve the above by increasing the number of cases, clarifying the problems, and investigating the factors for the reduction of 4F-PCC administration time.

## Conclusion

EICU pharmacist’s intervention can improve the process from 4F-PCC prescription to its administration and may reduce time to 4F-PCC administration.

The main content of this paper was presented at the 47th Annual Meeting of the Japanese Association for Acute Medicine (Tokyo, 2019).

## Data Availability

All data generated or analyzed during this study are included in this published article.

## Abbreviations

4F-PCC: Four-factor prothrombin complex concentrate
ASHP: American Society of Health-System Pharmacists
DIC: Disseminated intravascular coagulation
ED: Emergency department
EICU: Emergency intensive care unit
PT-INR: Prothrombin time-international normalized ratio
